# “Tobacco dependence treatment makes no sense because”…: Rebuttal of commonly-heard arguments against providing tobacco dependence treatment in the hospital setting

**DOI:** 10.1186/1471-2458-14-1182

**Published:** 2014-11-19

**Authors:** James Balmford, Jens A Leifert, Andreas Jaehne

**Affiliations:** Präventionsteam (PT), Tumorzentrum Freiburg, Albert-Ludwigs-Universität Freiburg, Hugstetter Str. 55, 79106 Freiburg, Germany; Department of Internal Medicine, Breisgauklinik, Bad Krozingen, Germany; Department of Psychiatry & Psychotherapy, Universitätsklinikum Freiburg, Freiburg, Germany

**Keywords:** Tobacco dependence treatment, Hospital, Tobacco control

## Abstract

**Background:**

The provision of tobacco dependence treatment in health care settings, particularly in countries lacking a history of strong tobacco control policy implementation, is limited by continued misconceptions on the part of health professionals and decision-makers regarding its worth and efficacy. In this paper, we rebut 9 arguments against the provision of tobacco dependence treatment that we have encountered in our experiences implementing and maintaining a dedicated smoking cessation service at a large university hospital in southern Germany.

**Discussion:**

Broadly, the arguments relate to the nature of addiction, the efficacy and safety of stop-smoking medication and behavioural support, and the benefits and challenges of quitting. They include: (a) If smokers really want to quit, they will be able to do it alone (without help); (b) You can’t forbid patients from doing what they want; (c) Patients will be upset if you talk to them about their smoking; (d) Stop-smoking medication has side effects that are more dangerous than smoking; (e) You have to be well trained to help smokers to quit (otherwise you can do more harm than good); (f) If you smoke yourself, you lack credibility; (g) If you have cancer, it is too late to quit; (h) Nicotine withdrawal is dangerous for heavy smokers; and (i) Smokers die earlier, thus reducing costs to the health system.

**Summary:**

It is hoped that the counter-arguments presented here arm tobacco control advocates and practitioners working in health care settings, particularly in countries which have not prioritised tobacco control, to respond appropriately and convincingly to those opposed to the provision of tobacco dependence treatment.

## Background

More than 5 million people are estimated to die each year from smoking related diseases worldwide [1]; it has been conservatively estimated that smoking kills about one half of all persistent users [2]. Quitting smoking has immediate as well as long-term health benefits for men and women of all ages, reducing risks for diseases caused by smoking and improving health in general [3], including among those with chronic disease [4, 5].

The health care system, including hospitals, is an important channel for delivering tobacco dependence treatment (TDT) to smokers. There is increasing recognition that treating tobacco use should be a high priority for health professionals and those who fund health care provision (e.g. [6]), and that all patients should be asked about tobacco use, advised to quit, and given appropriate assistance both during the hospital stay and post-discharge [6]. However, provision of TDT remains lower than optimal in many countries [7], including in Germany where we are located, where few hospitals provide structured cessation assistance to patients who smoke [8].

Moreover, the role of health professionals in providing TDT in Germany is often disputed [9]. Resistance to the provision of TDT may largely stem from misperceptions about the safety and/or efficacy of established treatments, and a lack of understanding of the nature of addiction and how this perpetuates tobacco use [9]. A recent study of over 19,000 medical students in Germany found that over half believed that willpower alone was more effective than a combination of group therapy and nicotine replacement therapy [10].

In this paper, we discuss and rebut nine of the most common arguments we have encountered in implementing a dedicated tobacco dependence treatment service at a large university hospital in Freiburg (a medium-sized city in southern Germany), and in our efforts to implement similar services in other hospitals throughout the country over the past couple of years. Broadly, these misperceptions relate to the nature of addiction (3 arguments), the efficacy and safety of stop-smoking medication and behavioural support (3 arguments), and the benefits and challenges of quitting (2 arguments). The final argument relates to the worth of quitting for the health care system, and being more philosophical in nature, we dispute it both on philosophical and empirical grounds.

For many in Western countries that have made considerable progress in tobacco control, the issues presented and arguments raised may seem rather basic, even irrelevant. However, our audience is not advocates in these countries, but rather those from countries which have not prioritised TDT for whom these issues are likely to be highly relevant. We hope to prepare advocates of TDT in health care settings for some of the counter-arguments they may also encounter, and more broadly to draw attention to the continuing knowledge gaps that lead to their perpetuation.

## Discussion

1. **Misperceptions related to the nature of addiction**
  - **If smokers really want to quit, they will be able to do it alone (without help).**
    Perhaps the most remarkable observation about tobacco use is the fact that many smokers simply do not want to continue to smoke. An international cohort study of smokers found that about 90% regret ever having started to smoke [11], and a similar proportion report that they would like to quit if it were ‘painless’ to do so [12]. Quit attempts are common - it is estimated that on average smokers make one serious quit attempt per year [13], but most attempts are unaided [14], and only 3-5% of unaided attempts are successful [15]. Older smokers [16, 17] and those who are more nicotine dependent [18] are less likely to make quit attempts, less confident in their ability to succeed, and more likely to experience regret.
    At the core of this belief is the perception that ‘really wanting to quit’ (i.e., high motivation) is both necessary and sufficient to be able to quit successfully. Seventy percent of smokers believe this to be the case [19]. However, while motivation to quit is a strong predictor of making a quit attempt, it is not predictive of success [20]. Indeed, some studies have found that smokers who are highly motivated to quit may even be less likely to succeed than those who are only moderately motivated [21, 22]. There are a number of available forms of behavioral or pharmacological tobacco dependence treatment that may improve a smoker’s chances of quit success. For example, a recent population-based study found that smokers are around four times more likely to succeed in quit attempts if they use stop-smoking medication, with one medication, varenicline, associated with a nearly sixfold increase in success [23]. Many smokers are either unaware of, or underestimate the benefit of, these effective forms of help [24].
  - You can’t forbid patients from doing what they want.
    People have a free choice to smoke, but free choice involves having adequate knowledge relevant to that choice (something that is central to the notion of informed consent). Health professionals, far from compromising their patients’ free choice, are actually enhancing their capacity to exercise free choice by ensuring that they understand how continuing to smoke may affect their health [25]. While almost all smokers know that smoking is harmful to health, few have a realistic understanding of either the severity of smoking-caused disease or their personal susceptibility [26]. Moreover, it could be argued that because of the addictive nature of nicotine, many smokers are not actually ‘doing what they want’ in continuing to smoke. Rather, as we have seen, many are continuing to engage in a behaviour that they know is harmful and which they would like to give up [11, 12].
    It is common for health professionals to recommend that patients modify their behavior when they believe such changes will be beneficial (for example, when patients are asked to reduce their weight). Providing patients with relevant information, and trying to motivate them to change their behaviour in ways that will benefit their health is consistent with providing quality health care.
  - **Patients will be upset if you talk to them about their smoking.**
    Few smokers proactively seek personal assistance to stop smoking. It is estimated that only 1% of smokers access telephone support services (quitlines) annually [27], and even fewer seek face-to-face counseling [14]. Nonetheless, a far higher proportion of smokers are interested in smoking cessation advice and support if it is offered to them [28–32]. For example, when smokers were offered an Internet or mobile phone-based automated intervention without prior knowledge that this would occur, 60% accepted the offer, and most of these went on to use the intervention sufficiently to obtain a probable therapeutic benefit [30]. In a primary care setting, simply informing smokers by letter that they could access a free consultation with a tobacco treatment specialist resulted in over 10% taking up the offer [31]. Finally, over 80% of patients referred to the smoking cessation service we established at the Universitätsklinikum Freiburg agreed to receive counseling when approached at bedside, and of these, over half (55%) agreed to formulate a treatment plan including the receipt of post-discharge phone support [32].
    When offered support in an empathic, respectful manner, it is possible to reach even those smokers who appear unmotivated to quit [33]. The principles of motivational interviewing [34] provide guidance on effective ways to understand smokers’ attitudes and beliefs and to increase their motivation to quit.
    Patients who smoke typically appreciate offers of cessation assistance, despite not always appearing to respond favourably. For example, in an intervention study conducted in a German hospital, two-thirds of cardiology patients offered brief smoking cessation counselling reported that the counselling was helpful, and 87% said that it was important to them. Interestingly, this contrasted markedly with qualitative reports of nurses’ perceptions of the same interactions, which were that patients often felt uncomfortable and refused to be counselled [35]. Health professionals should be encouraged that their efforts to address their patients’ smoking are likely to be well received.
2. **Misperceptions related to the efficacy and safety of stop-smoking medication and behavioural support**
  - **Stop-smoking medication has side effects that are more dangerous than smoking.**
    Nicotine replacement therapy (NRT), available over-the-counter in many countries, is designed to alleviate symptoms of nicotine withdrawal [36]. NRT does not mimic the effects of a cigarette, but delivers low-level doses of nicotine in a non-combustible form and at a slower rate, and as such has low abuse potential [37]. NRT does not contain the over 4000 other chemicals present in tobacco smoke, of which 69 are known carcinogens [38].
    The safety and efficacy of NRT has been studied in clinical trials and real-world studies for over 30 years. A systematic review of clinical trials found that NRT is associated with minor adverse events, including insomnia (11.0%) and gastrointestinal complaints (10.8%), which may cause mild discomfort [39]. The most serious adverse event reported with notable frequency was heart palpitations/chest pains (3%). Safety concerns have been expressed for people with pre-existing cardiovascular disease, but there is no evidence that NRT causes life-threatening complications (see also [40]), and the benefits of smoking cessation exceed any risk [41]. In addition, there is currently insufficient evidence for the safety or efficacy of NRT in pregnancy [42].
    Other common side effects of NRT are associated with the mode and site of administration (e.g. 0 skin irritation where the nicotine patch is placed, or mouth ulcers following use of orally-administered NRT). Such problems are generally mild and can be easily overcome by switching to a different form (e.g. from the patch to nicotine gum).
    Two forms of prescription-only medication for smoking cessation, bupropion and varenicline [43, 44], are associated with some additional safety concerns. Bupropion is associated with an increased risk of seizure, occurring in 0.1% of users [43], and is consequently contraindicated in smokers with a seizure disorder. A 2011 meta-analysis reported an association between varenicline use and increased cardiovascular events [45], leading the US Food and Drug Administration (FDA) to issue a warning that varenicline might increase the risk of cardiovascular events in patients with cardiovascular disease [46]. However, this review has been criticised for inappropriately excluding trials with no reported adverse events, among other methodological issues. A more recent review found no significant increase in serious cardiovascular adverse effects associated with varenicline use, either during treatment or for 30 days post-treatment [47].
    Concerns have also arisen about the potential of bupropion and varenicline to exacerbate neuropsychiatric symptoms. A full discussion of this issue is beyond the scope of this article, but the FDA currently recommends that both medications be used with caution by individuals with a history of psychiatric illness [46].
  - **You have to be well trained to help smokers to quit (otherwise you can do more harm than good).**
    Clinicians can do a number of things to encourage their patients to quit that do not require extensive training. At any given time, even though most smokers are not actively planning to quit, thoughts about quitting are common [13]. Thus, the task is often to encourage smokers to commit themselves to making the quit attempt that they are contemplating, rather than to convince them to do something they do not want to do.
    Research has shown that even brief advice delivered by a physician (of as little as three minutes duration) can be effective compared to no advice, increasing quitting by 1-3%, and effectiveness increases with greater duration [48]. While this may not seem to be a large difference, and it is easy to get discouraged by a perceived lack of success, motivation to quit can come from numerous sources (e.g. friends, family, policies designed to reduce tobacco use, and health professional advice). Each of these influences is likely to have a cumulative effect.
    Clinicians can easily learn to administer very brief, effective advice and assistance by obtaining minimally intensive training [48]. However, those who do not feel confident about their ability to provide more extensive behavioral support, where patients need it, usually have a number of treatment options to which smokers can be referred. Behavioral support designed to enhance motivation and teach practical skills and strategies can be provided by telephone quitlines, qualified therapists or via new technologies such as the Internet or mobile phones. Simple referral mechanisms to these services are frequently available. Clinicians will benefit from familiarising themselves with reputable websites (http://www.quitcoach.org.au and http://www.smokefree.gov are good examples; high-quality German-language sites include http://www.bzga.de und http://www.tabakkontrolle.de), and the number of the local quitline.
  - **If you smoke yourself, you lack credibility.**
    First, it is important to acknowledge that this is a genuine issue. It is likely to be difficult for health professionals who smoke to convince patients to make lifestyle changes that they themselves are not making. Accordingly, primary care physicians who smoke are less likely to discuss smoking with their patients or to provide quitting assistance [49].
    However, it is not hypocritical for a doctor who smokes to advise a patient that smoking can damage his or her health. Quitting smoking is unequivocally a good thing for the patient’s current and future health, regardless of the smoking status of the person giving the advice. It is of course possible that some smokers will reject advice from a physician who smokes because they perceive him/her as lacking credibility. On the other hand, some may be more likely to accept advice from a physician who has personal experience of the difficulty of quitting. We know of no studies that have explored this, but clinical experience suggests that many smokers want to know whether the person advising them to quit has ever smoked.
3. **Misperceptions related to the benefits and challenges of quitting**
  - **If you have cancer, it is too late to quit.**
    Cancer patients who quit smoking experience the many physical and psychological benefits of quitting that are common to all patients, outcomes that may contribute in turn to improved response to treatment. Continuing to smoke following a cancer diagnosis contributes to a number of adverse outcomes, including tumour progression, the development of a second primary cancer e.g. [50] and poorer survival rates following chemotherapy treatment e.g. [51]. Smoking is also known to negatively affect the ongoing quality of life of cancer patients [52, 53]. Even in advanced lung cancer, quitting smoking is known to improve pulmonary function [54].
    A cancer diagnosis is a major motivator for a quit attempt. Reviewing 11 studies that reported rates of abstinence after a cancer diagnosis, Cox et al. [55] found that across studies, 14-58% of patients who smoked at the time of diagnosis continued to smoke after cancer treatment. This compares favourably to annual quit rates of 4-5% in the general population of smokers [56], yet many cancer patients continue to smoke. At all stages in the cancer treatment process (diagnosis, during oncological treatment, post-treatment recovery), cancer patients are typically receptive to advice about quitting smoking and to participation in tobacco dependence treatment [55]. Cox et al. [55] stresses the need for interventions which are sensitive to the needs of cancer patients, both to promote initial abstinence and to prevent smoking relapse.
  - **Nicotine withdrawal is dangerous for heavy smokers.**
    Common symptoms of nicotine withdrawal include cravings, irritability/anger/frustration, anxiety, depression, impaired concentration, insomnia and restlessness [36]. Many of these are common to a number of other drug withdrawal syndromes, not only to smoking cessation. While the time course of the individual symptoms tends to differ, nicotine withdrawal peaks on average within the first week of abstinence and lasts for 2–4 weeks.
    It is difficult to know in what sense nicotine withdrawal is considered dangerous by those ascribing to this view. Certainly, impaired concentration or insomnia could lead to accidents, but we are unaware of any medical basis for the argument. In smokers with a mental health condition, e.g. depression, smoking cessation may require medical management due to required changes in medications affected by nicotine [57].
    Nicotine replacement therapy can reduce the severity of withdrawal symptoms, easing physical discomfort and controlling urges to smoke [58]. Smokers who have had the experience of temporary withdrawal, e.g. during long-distance flights or when suffering from a cold and unable to smoke, have experience coping with these symptoms at least for a while. It should also be noted that the experience of severe withdrawal symptoms is not a given; many ex-smokers report that quitting smoking was easier than they expected [59].
    To the extent that hospitals implement smoking restrictions, even patients who do not want to quit may experience some degree of nicotine withdrawal. For others, nicotine withdrawal may be a primary concern. The nicotine patch is considered the most appropriate form of NRT for hospitalized patients if they are experiencing nicotine withdrawal [60], because unlike oral forms it does not require repeated administration throughout the day. It has been recommended that patients who smoke more than 10 cigarettes per day should be provided with NRT if not contraindicated [61].
4. **Smokers die earlier, thus reducing costs to the health system.**
  It is often argued that smokers actually save the health system money by dying early, thereby avoiding lengthy and expensive care in old age. One of the first studies to have explored whether this is true, published in the New England Journal of Medicine [62], found that because non-smokers live longer and incur greater costs of treating diseases unrelated to smoking, the initial savings on health care costs if all smokers quit would be reversed within 15 years. However, this study only considered a narrow range of smoking-related diseases, and is therefore likely to have greatly underestimated differences in health care utilization between smokers and non-smokers. A more recent Danish study [63] included a broader set of smoking-related diseases, and found substantial lifetime cost savings resulting from smoking cessation, particularly in those who quit at younger ages (e.g., €24,800 in total lifetime healthcare costs in a moderate smoker, male, who quits at age 35).
  Studies which conclude that smoking has a net economic benefit to a society tend to be beset with methodological flaws, most notably (a) counting tobacco taxes as benefits of smoking, when they are nothing more than a transfer of resources already in the economy (i.e., the same benefit would accrue if taxes were levied on alternative goods and services); and (b) failure to conduct the appropriate comparison: the economy with tobacco versus the economy without tobacco [64]. Were smoking prevalence to reduce to 0%, expenditure previously allocated to tobacco would simply be reallocated, with the resultant growth in other industries making up for (or adding to) any economic effect of reduced smoking. A full discussion of this and other economic arguments has been provided by Warner and Fulton [65].
  Notwithstanding the economic benefit of reduced tobacco consumption, the notion that early, avoidable deaths should be considered a virtue is highly troubling from an ethical perspective, particularly when this argument is put forward by those who have a professional duty to prolong life. Each person who dies in middle age as a result of smoking is just that: a person; someone’s mother or father, best friend, or valued work colleague. It is also fails to recognise the reality of long-term reduced quality of life that is characteristic of chronic smoking-related diseases such as emphysema (which naturally result in greatly increased health care expenditures during smokers’ lives).

## Summary

In this paper, we have presented 9 arguments which may be used to oppose the provision of tobacco dependence treatment in the hospital setting, typically in countries lacking a history of strong tobacco control policy and/or where the health care system has not prioritised tobacco treatment delivery. It is hoped that the counter-arguments presented here anticipate these concerns and provide advocates with ways to respond convincingly.

The importance of using the opportunity afforded by hospitalization to help smokers end their dependence on tobacco should not be underestimated. Hospitalization provides a unique opportunity to help smokers initiate a smoking cessation attempt, and receive the support they need to ensure that the attempt is successful. It is a potentially powerful ‘teachable moment’ in which smokers are often particularly motivated to quit and receptive to assistance due to concerns about their health [61]. We echo Fiore’s [6] endorsement of the US Joint Commission’s Tobacco Cessation Performance Measure-Set, which mandates participating hospitals to deliver evidence-based tobacco dependence counselling and medication for all admitted patients who use tobacco, and promote it as a model for implementation in other countries. Health care systems should also prioritise efforts to train health professionals in tobacco dependence treatment, so that brief counseling of smokers becomes part of their daily routine, and to increase awareness of the various forms of evidence-based treatment. Moreover, there is a need to provide accessible and effective smoking cessation assistance to health professionals who smoke themselves, so they do not undermine their status as communicators of health information and role models for healthy behaviour.

In addition, we have argued that the majority of hospitalised smokers, despite possible ambivalence toward stopping smoking, will nonetheless be receptive to (and often grateful for) the offer of non-judgmental assistance to quit. It is common for health professionals to feel ill-equipped to provide such assistance, but even very brief advice can have a positive impact. Where more intensive help is desired, a number of safe and effective stop-smoking medications exist, as well as a range of behavioural treatment options. We caution, however, that discharged patients will rarely actively make contact with cessation services to which they have been referred [66]. For this reason, if contact with a face-to-face or telephone service is recommended, it is likely to always be preferable for the service to initiate contact with the patient (where possible). In countries where telephone counselling is widely available, hospitals and quitlines can work collaboratively to ensure that post-discharge support is provided proactively to smokers [67].

Finally, we have argued against the presumption that if smokers really want to quit, they will be able to do so without help. High motivation to quit smoking does not inevitably lead to success. However, this is not the same as arguing that smokers are unable to quit without help. Indeed, most successful ex-smokers report having quit unassisted; it is by far the most widely-used strategy [59]. We recommend that health professionals respect their patients’ wishes if they prefer to try to quit without formal help, but to monitor their progress, normalise difficulties, and be ready to discuss treatment options if unassisted attempts fail.

## Abbreviations

TDT: Tobacco dependence treatment
NRT: Nicotine replacement therapy
FDA: Food and Drug Administration.
